# Indirect orthodontic bonding - a modified technique for improved efficiency and precision

**DOI:** 10.1590/2176-9451.20.3.109-117.sar

**Published:** 2015

**Authors:** Lincoln Issamu Nojima, Adriele Silveira Araújo, Matheus Alves

**Affiliations:** 1 Adjunct Professor, Universidade Federal do Rio de Janeiro (UFRJ), Department of Orthodontics, Rio de Janeiro, Rio de Janeiro, Brazil; 2 PhD resident of Orthodontics, Universidade Federal do Rio de Janeiro (UFRJ), Rio de Janeiro, Rio de Janeiro, Brazil; 3 MSc in Orthodontics, Universidade Federal do Rio de Janeiro (UFRJ)

**Keywords:** Dental bonding, Orthodontic brackets, Orthodontic devices, Corrective Orthodontics

## Abstract

**INTRODUCTION:**

The indirect bonding technique optimizes fixed appliance installation at the orthodontic office, ensuring precise bracket positioning, among other advantages. In this laboratory clinical phase, material and methods employed in creating the transfer tray are decisive to accuracy.

**OBJECTIVE:**

This article describes a simple, efficient and reproducible indirect bonding technique that allows the procedure to be carried out successfully. Variables influencing the orthodontic bonding are analyzed and discussed in order to aid professionals wishing to adopt the indirect bonding technique routinely in their clinical practice.

## Abstract (pt)

**INTRODUÇÃO:**

a técnica de colagem indireta prioriza a otimização do procedimento de montagem do aparelho fixo na clínica ortodôntica, assegurando, entre outras, vantagens relacionadas à precisão no posicionamento dos braquetes. Nesse procedimento clínico laboratorial, o material e o método de confecção da moldeira de transferência são determinantes no quesito precisão.

**OBJETIVO:**

este artigo descreve uma técnica de colagem indireta simples, eficiente e reprodutível, para que o procedimento possa ser realizado com sucesso. Variáveis que exercem influência sobre o procedimento são analisadas e discutidas, a fim de auxiliar o profissional a adotar, de forma rotineira, a técnica de colagem indireta em sua prática clínica.

## INTRODUCTION

All orthodontists share the goal of achieving excellent results when clinically treating patients. Despite its complexity, treatment success relies on correct positioning of brackets during bonding, which will simplify subsequent phases of orthodontic treatment in addition to increasing predictability of results.^1^

In this context, the indirect bonding technique stands out for allowing better three-dimensional visualization of tooth positioning and, as a result, greater accuracy while positioning brackets,^2^ since the procedure is carried out in the laboratory, followed by transference to the patient's mouth by means of custom-made trays. This advantage was confirmed by Hodge et al^3^ who found that errors associated with bracket positioning were minimized when indirect bonding was chosen over direct bonding, under any of three aspects of observation: height, mesiodistal position and angulation. Better height positioning was also observed by Koo et al,^4^ while Aguirre et al^5^ emphasized the higher technical precision provided by the indirect technique used for brackets angulation on maxillary and mandibular canines and height positioning on maxillary canines.

Other advantages associated with indirect bonding are: (1) reduced chair time,^1^^,^ 2 ^,^ 6 (2) little need for compensation bends,^1^ (3) reduced physical and mental stress, since the clinical procedure is simpler than direct bonding,^1^ and (4) more comfort for the patient.^6^^,^ 7

Disadvantages such as time-consuming laboratory procedures and additional costs with material are overcome by the previously stated benefits, which ends up propagating the technique. As a result of the growing popularity of indirect bonding, new techniques^8^ have been developed. These techniques stand out especially on the bonding system applied (self 8 or light-curing^9^) and the transfer tray used (hot glue,^10^ addition silicone,^1^vacuum-formed,^8^ prototyped^11^ or associated methods^12^). Despite the variety of techniques proposed, indirect bonding is not considered a gold-standard procedure yet, probably due to the numerous variables inherent to the process and which need to be controlled if success is to be obtained.^12^

The improvement of the technique in order to yield better clinical results is the aim of the various modifications that have been suggested. Assuming the same precept, this article describes a new method of indirect bonding that has showed precision in bracket placement and efficiency in orthodontic bonding as the end result. The technique will be described herein in detailed steps, encompassing clinical and laboratory stages. 

## INDIRECT BONDING TECHNIQUE

All steps involved in indirect bonding are divided into three stages: Clinical Stage I, Laboratory Stage and Clinical Stage II. 

## Clinical Stage I

1. Perform dental prophylaxis and upper and lower full-arch impressions with high quality alginate, following the manufacturer's instructions. Examine in full detail the impression obtained, in order to avoid potential flaws that may lead to distortions in the dental cast, paying special attention to the areas corresponding to teeth. 

2. Obtain dental casts with type IV dental stone. This procedure should be carried out judiciously so that dental casts are free from imperfections (positive and negative bubbles). Surface flaws will hinder brackets and tray fitting to the teeth, when the former are transferred to the oral cavity. It is also necessary to wait for the stone to fully crystallize and dry. 

## Laboratory Stage

**Figure 1.**
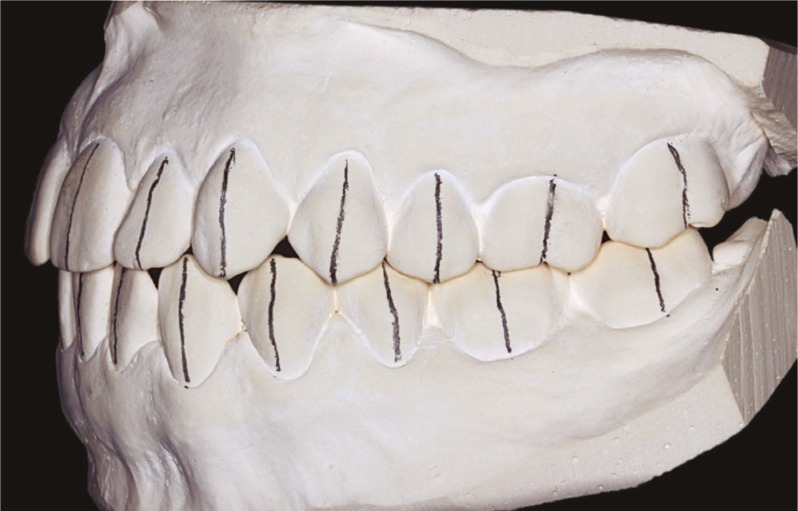
Long axes of teeth marked in black pencil.

**Figure 2.**
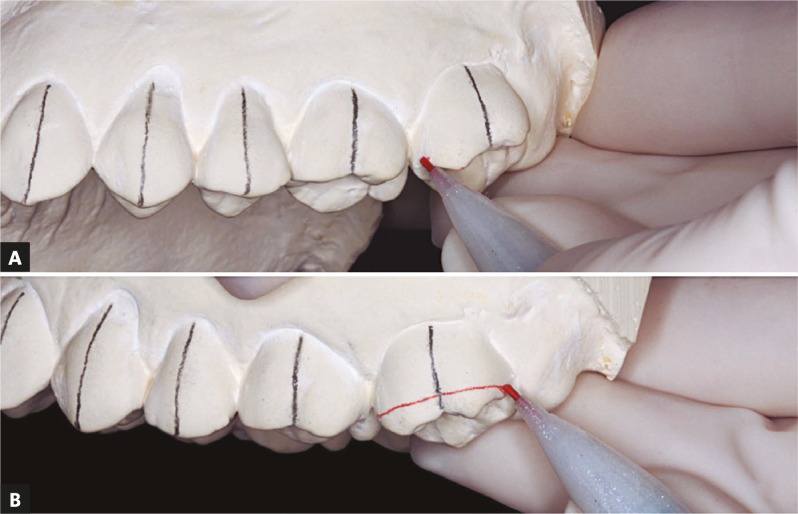
A) Mark the mesial and distal marginal ridges with red pencil on buccal surface of teeth. B) Join the marked points, determining the height of marginal ridges.

**Figure 3.**
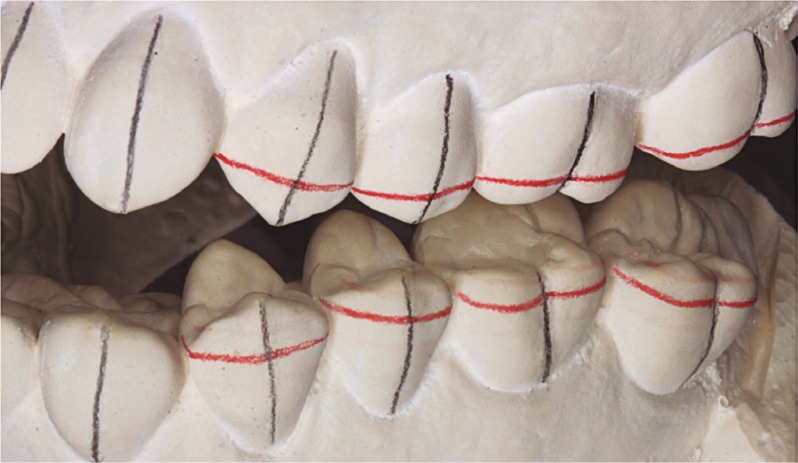
Marginal ridge heights marked on all posterior teeth.

**Figure 4.**
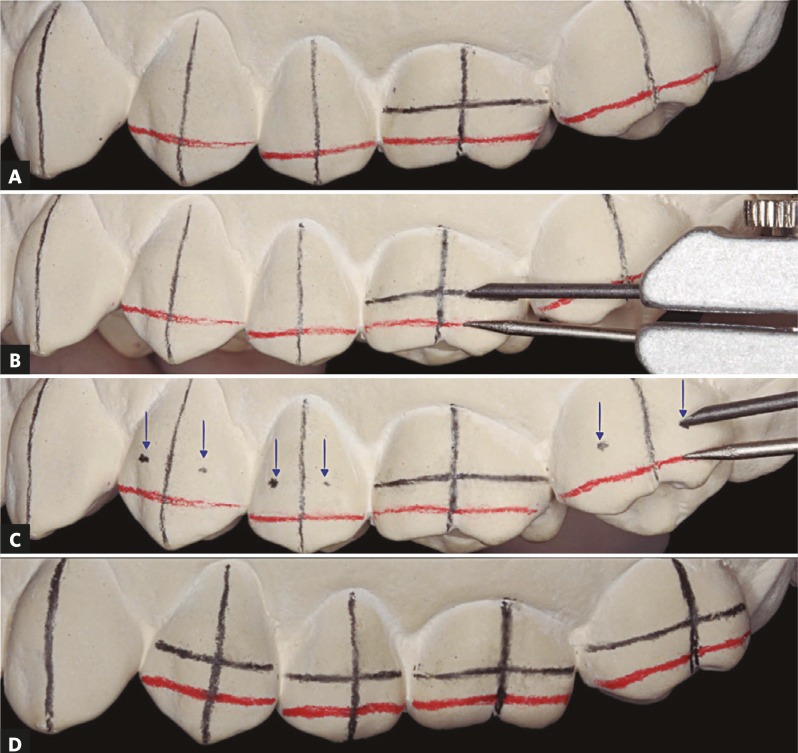
A) First molar slot height determined in black pencil using a bracket placement marker gauge. B) Measurement of distance between two horizontal lines on the first molar with a drawing compass. C) Reproduction of the same distance on buccal faces of other posterior teeth (blue arrows). D) After joining the marked points, premolars and molars slot heights are determined.

**Figure 5.**
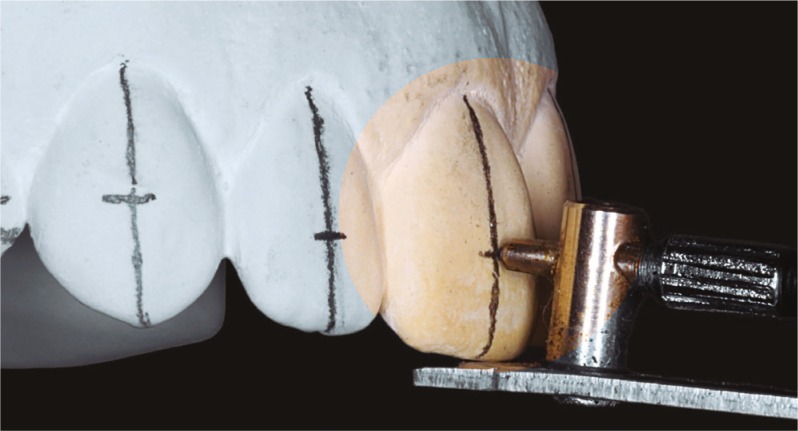
Plan vertical position of incisor and canine brackets and transfer to the cast using a bracket placement marker gauge.

**Figure 6.**
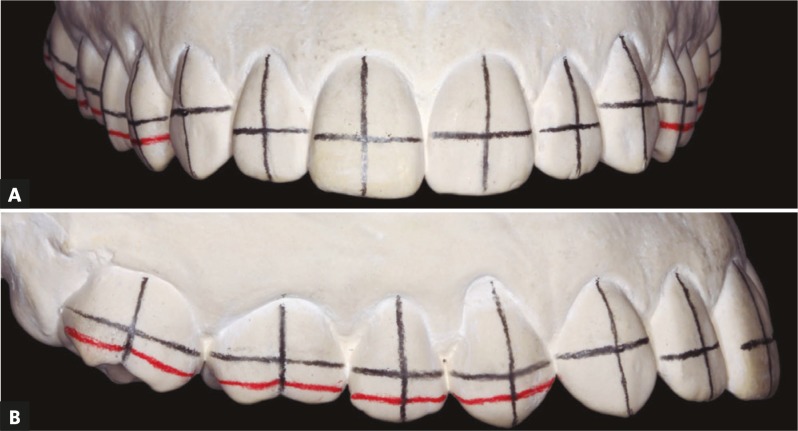
Final aspect of bracket bonding guide in A) frontal and B) lateral views.

**Figure 7.**
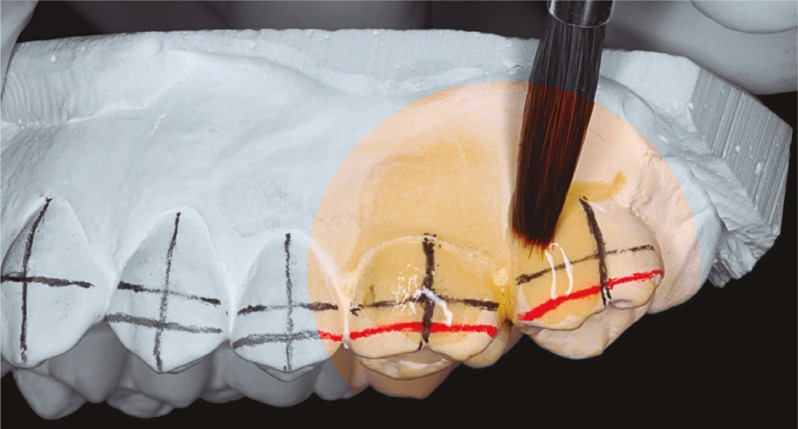
Application of separator diluted in 1:1 water ratio.

**Figure 8.**
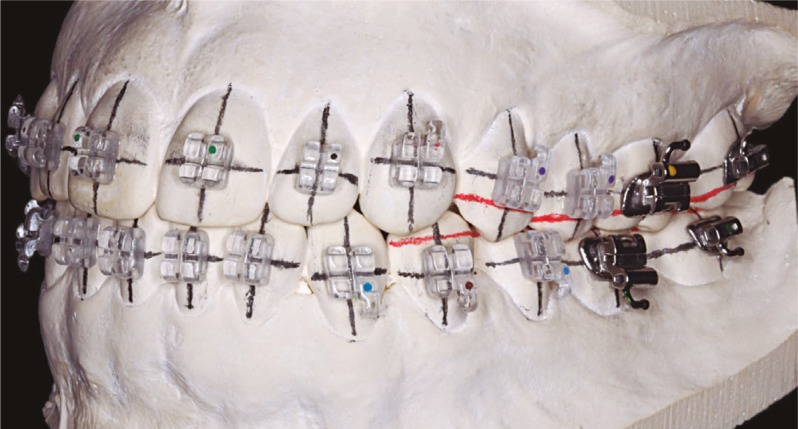
Bracket bonding with light-curing adhesive over drawn guide lines, respecting slot height and the long axis of each tooth.

**Figure 9.**
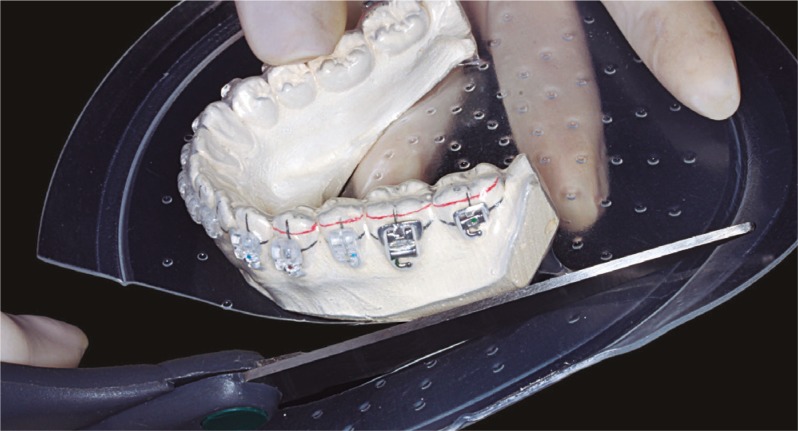
Trim excess 1 mm-Ethylene-Vynil Acetate (EVA)-soft tray.

**Figure 10.**
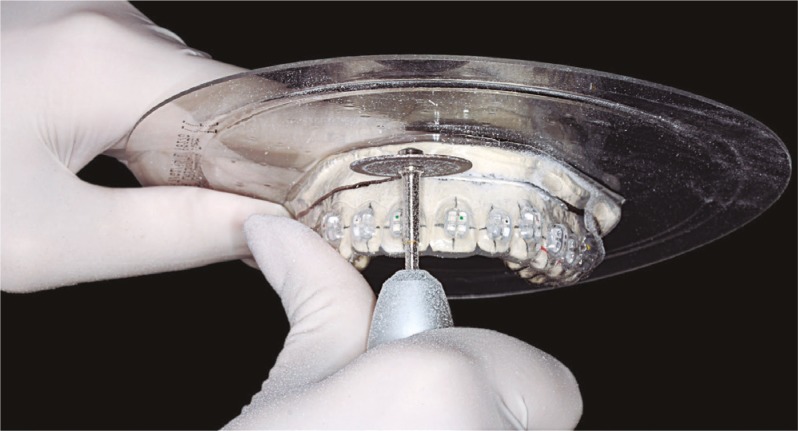
Trim Cristal and Soft trays with a carborundum disk, 2 to 3 mm above the cervical margin, on both buccal/labial and palatal surfaces.

**Figure 11.**
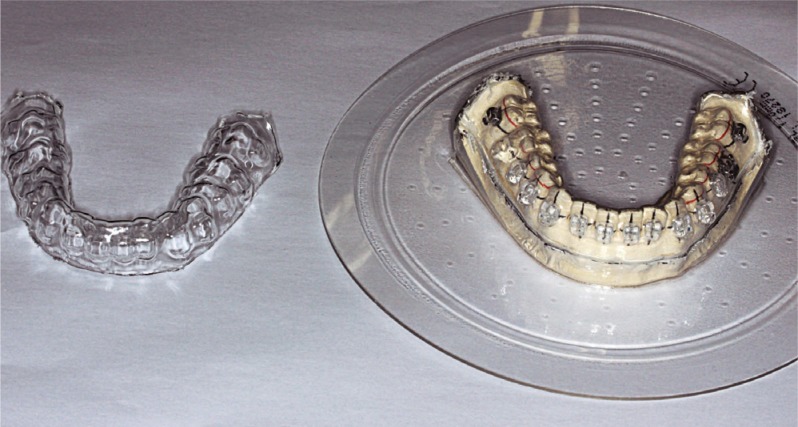
Cristal tray separated from Soft tray and cast.

**Figure 12.**
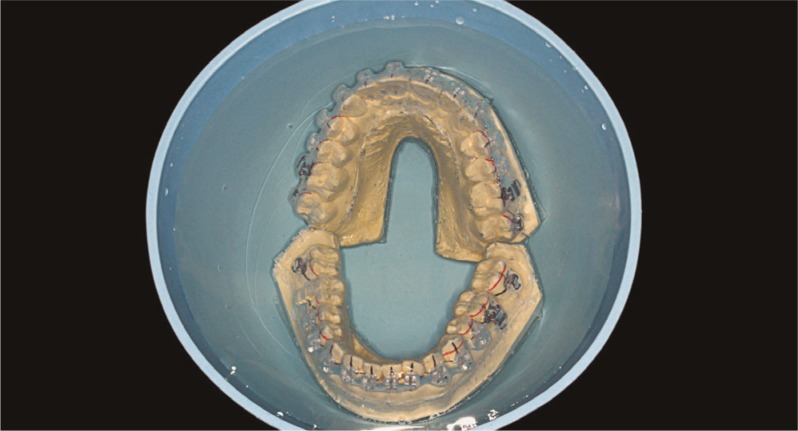
Soft tray and cast immerged in water to dissolve the separator after trimming excess of Cristal tray.

**Figure 13.**
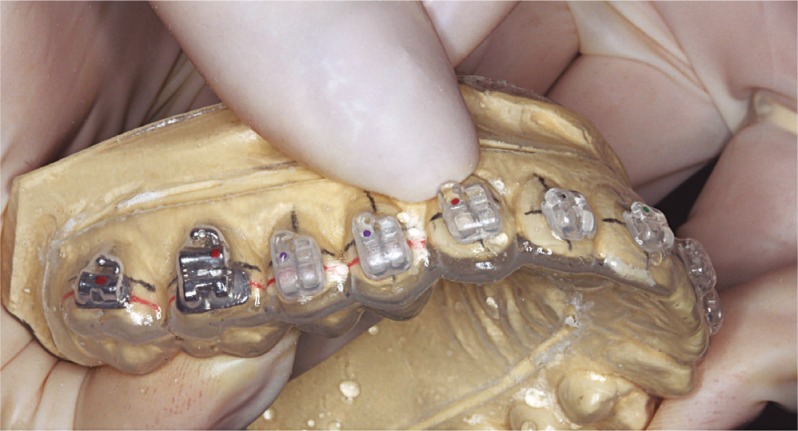
Apply digital pressure over each bracket de dislodge it from cast surface.

3. Draw bracket positioning guidelines on the previously obtained cast. First, with the aid of a black pencil, determine the long axis of each tooth on the center of its crown, using a panoramic radiograph as an auxiliary method to observe tooth angulation and increase accuracy (Fig 1). With the aid of a red pencil, mark the projection of mesial and distal marginal ridges on the buccal surface of premolars and molars, then join the two points (Figs 2A and 2B). Horizontal red lines represent the height of posterior teeth marginal ridges and establish the depth of occlusal contact. This procedure should be repeated for all posterior teeth (Fig 3). Draw bracket slot height using a black pencil, starting from the first molar (Fig 4A). This position depends on the type of malocclusion and on the anatomical shape of teeth. In open bite and hyperdivergent faces, brackets should be placed closer to the occlusal surface of teeth; that is, close to the red line, thus avoiding teeth extrusion, which could compromise treatment results. On the other hand, in deep overbite malocclusions, when extrusion of posterior teeth is necessary, brackets should be placed slightly further from the red horizontal line. 

4. With the aid of a drawing compass, determine the distance between the two horizontal lines in the first molar (Fig 4B) and replicate it on the buccal surfaces of other remaining posterior teeth (Fig 4C), thus establishing bracket slot height (Fig 4D). 

5. Calculate and transfer the slot height of incisors and canines to the cast using a bracket placement marker gauge (Fig 5). Reference tables can be used to determine bracket height of anterior teeth, according to the type of vertical malocclusion. In open bite cases, brackets can be placed more gingival on incisors and canines; whereas in deep bite malocclusions, they can be placed slightly closer to the incisal edges. In most cases, we recommend placing the canine bracket at the same height as the first premolar, measured from the slot to the cusp tip. For lateral incisors, subtract 1 mm from canines height, and for central incisors, add 0.5 mm to lateral incisors height (Fig 6). 

6. Treatment plan should be reviewed with casts in occlusion, and brackets previously selected prior to drawing the guide lines on the lower cast, so as to avoid setbacks during definitive bonding, such as lower brackets interfering in postbonding occlusion. 

7. Apply a thin layer of separator (Cel-Lac; SS White, Rio de Janeiro, RJ, Brazil), mixed with water in a 1:1 ratio, over cast teeth surfaces. Brush the material in the same direction and wait for at least 20 minutes for it to dry completely (Fig 7). 

8. Apply orthodontic light-curable adhesive (Transbond; 3M Unitek, Monrovia, California, USA) to the bracket base and position it over the cast surface. Follow the previously established bonding guide, so that slot and long axis of brackets lie over the drawn guide lines. Press the bracket over the pre-established location and remove excess adhesive (Fig 8). Once all brackets were placed and positions were checked, use a light-curing unit, for example Triad 2000 system (Dentsply, York, PA), to cure the adhesive according to the manufacturer's instructions. Should this type of unit be unavailable, use conventional light-curing devices, directing the beam towards the mesial and distal sides of each bracket, for 15 seconds each and at 2 to 3 mm distance. 

9. Manufacture the transfer tray. Using a vacuum former, thermoform a 1-mm thick sheet of Ethylene Vinyl Acetate (EVA-foam) (Soft; Bio-Art, São Carlos, SP, Brazil) over the cast. After heated, once the sheet reaches 10 to 12 mm of distortion, according to manufacturer's instructions, it is ready to be formed. Trim excess material with scissors (Fig 9) and spray a thin layer of silicone over the tray to help separate it later from the second tray, to be made with more rigid material. Thermoform a 1.5-mm thick sheet of Polyethylene Trephthalate Glycol (PETG-plastic) (Cristal; Bio-Art) and trim both plates using a carborundum disk, 2 to 3 mm above the cervical margin of teeth, on both buccal/labial and lingual/palatal surfaces (Fig 10).

10. Separate the Cristal tray from the set (Fig 11), trim its labial/buccal surface up to the gingival margin of bracket wings, eliminating retention. Use a Scotch Brite brush to finish it and rinse with water and soap. In the meantime, immerse the cast and the Soft tray in water for 15 minutes to dissolve the separator (Fig 12). Press delicately each bracket to dislodge it from the cast (Fig 13). Fit the Cristal tray over the Soft tray and remove them from the dental cast. Clean the Soft tray and the adhesive bases with water and soap, abrading them gently with an interdental brush, rinse and dry them completely with oil-free compressed air. Trim any excess of Soft tray material with scissors, without detaching it from the outer tray. 

11. After stone blasting on bracket bases for 2 seconds to remove residual separator, an opaque surface will form. It is recommended that stone blasting be carried out using 50-µm particle size aluminium oxide under light pressure. Additionally, special care should be taken not to excessively abrade the adhesive. Clean trays with oil-free compressed air. 

## Clinical Stage II

**Figure 14.**
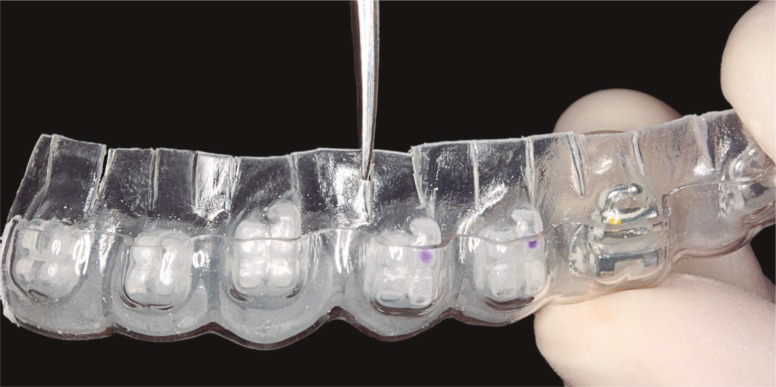
Vertical slits cut on Soft tray with sharp-point scissors above mesial and distal bracket wings

**Figure 15.**
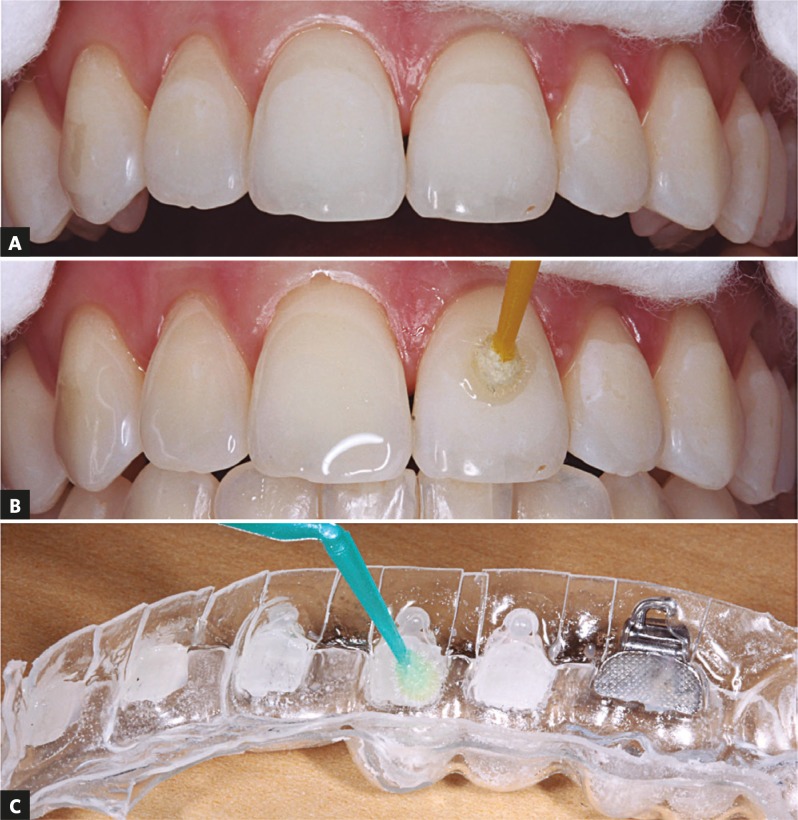
A) Acid-etching teeth surfaces after prophylaxis and B) applying ad-hesive. C) Application of a single layer of adhesive to the base of each bracket.

**Figure 16.**
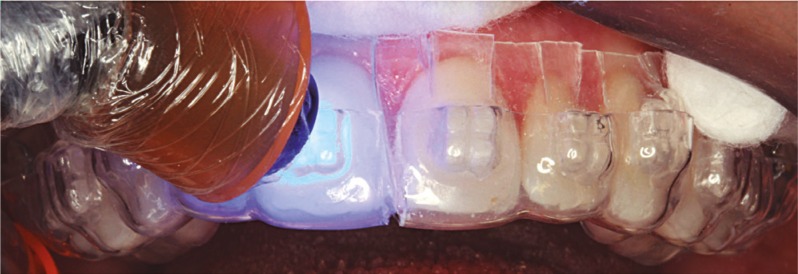
Fit the transfer tray to teeth without exerting too much pressure. Once confirmed the correct position through the clear tray, light-cure the adhesive.

**Figure 17.**
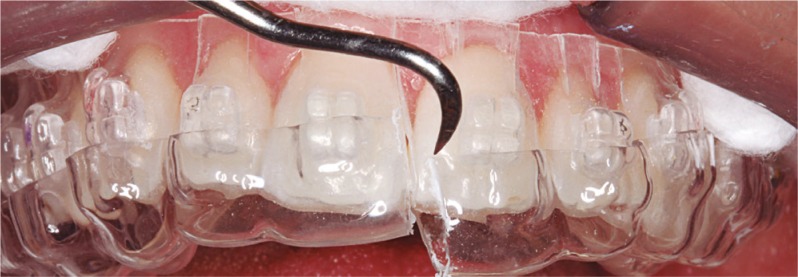
Remove the Cristal tray, pushing it in the occlusal direction

**Figure 18.**
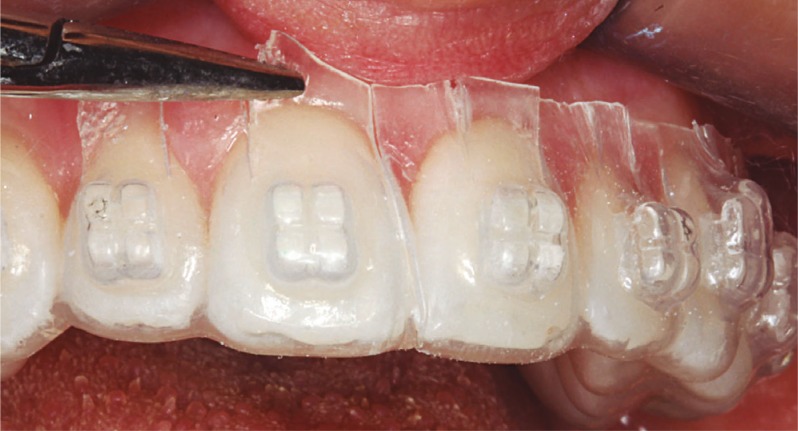
Remove the Soft tray. Use Mathieu pliers to pull off areas above the slits, liberating retention. Follow by completely removing the tray.

**Figure 19.**
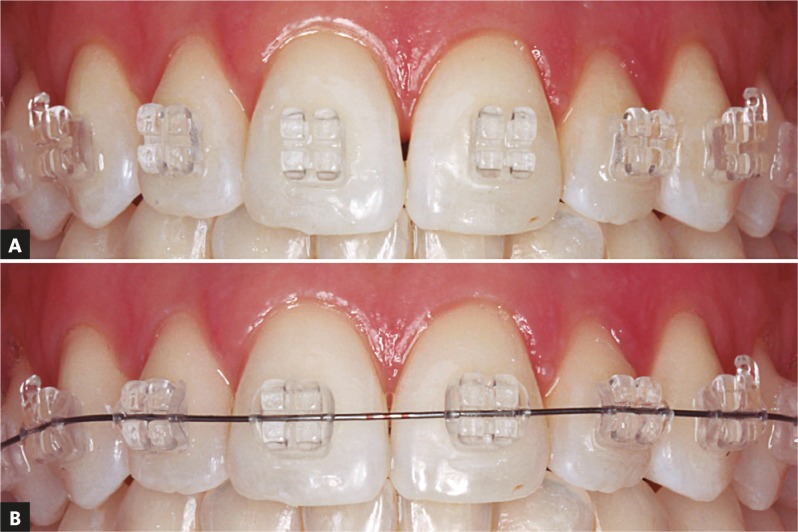
A) End of the indirect bonding procedure. B) Orthodontic archwires can be inserted into the slots.

12. Without detaching the trays, cut vertical slits on the Soft tray, above the mesial and distal bracket wings, using a sharp tip pair of scissors (Fig 14). This procedure will facilitate tray removal after bonding. Slits should be cut immediately prior to the clinical stage, to avoid undesired bracket displacement in between procedures, since they decrease tray retention. 

13. Perform prophylaxis using extra-fine pumice or oil-free paste, and etch teeth areas to be bonded with 37% phosphoric acid during 20 seconds. Wash, for additional 20 seconds, each etched surface (Fig 15A). 

14. Isolate area with cotton rolls and dry thoroughly. 

15. The decision whether to bond the full arch at once or in separate parts, by cutting trays into two or three segments, is influenced by the quality of isolation achieved and ease of insertion of the transfer tray. 

16. Select and apply adhesive to tooth surface and bracket base, following the manufacturer's instructions. Clinical experience and *in vitro*studies^13^^,^ 14 have demonstrated satisfactory results when Transbond XT Primer adhesive (3M Unitek) is used for direct orthodontic bonding. A thin layer of material should be applied to the etched tooth surface, followed by gentle air spray and reapplication (Fig 15A). A single application over bracket base should also be carried out (Fig 15B). 

17. Carefully position the tray over teeth. Once completely fitted, it is not recommended to exaggerate on the pressure to stabilize it. Visually confirm tray correct position through the clear tray and light-cure each mesial and distal bracket edges during 10 seconds (Fig 16) or use multiple tip light-curing devices for indirect bonding. 

18. Remove the firm Cristal tray with the aid of a smooth tip instrument, first pressing to dislodge it towards the occlusal edge (Fig 17). Use Mathieu pliers to pull the Soft tray off the previously slit areas above each bracket, releasing residual retentions (Fig 18), then fully remove the tray.

19. Remove cotton roll isolation and any excess adhesive with proper instruments. Should excess adhesive be noticed around brackets, use specific low-speed burs to remove it. Floss interproximal areas to secure they are clean. Orthodontic wires can be inserted immediately (Fig 19).

## DISCUSSION

The indirect bonding technique should: (1) provide high accuracy in bracket placement and (2) be of simple execution.^1^ Achieving success with the described technique is not complex, provided attention is paid to the recommended details. It allows precise orthodontic appliance installation in only one appointment, and can be used to place any bracket system commercially available. 

A critical factor concerning the indirect bonding technique is transferring brackets to teeth with precision and adequate bond strength,^15^ circumstance influenced solely by material selection and method of building up a transfer tray. The exclusive use of soft materials can result not only in imprecision in bracket positioning, but also in high incidence of bond failure as a result of poor fitting.^16^ An example of soft material is thermoplastic ethylene-vinyl acetate copolymer, in the form of a stick ("hot glue") or in thermoforming sheets. 

Faced with a variety of trays made up of different types of material, from silicone-based polymers (clear or opaque) to thermoplastic material,^10^^,^ 17 Castilla et al^16^ set out to compare *in vitro*, the accuracy of five types of transfer tray.^8^^,^ 18 ^,^ 19 ^,^ 20 Addition silicone trays displayed improved accuracy, but only in the occlusogingival plane. However, when average imprecision in height position were compared, values close to 0.095 mm were obtained for techniques employing silicone trays, against 0.12 mm for techniques using two thermoplastic trays, as described in this study. This difference found, besides being smaller than the method error used in the trial (0.07 mm), does not justify, in our critical evaluation, the preference for addition silicone. According to Koga et al,^18^ the technique using this material is sensitive, requires agility and efficiency during tray confection and is expensive. The greater complexity, material opacity, or even difficulty finding clear silicone within the local market, encourage the use of thermoformed trays. 

The proposed technique presents significant modifications when compared to the similar method evaluated by the aforementioned study.^16^Some of the amendments greatly improve accuracy in bracket placement. The gain in outer tray thickness and boundaries offers improved hardness and stability to the bracket transfer system. At this moment, it is highly recommended that trays be only lightly fitted, without application of additional force to stabilize them, which could cause deviations in ideal bracket positioning. Differences in placement accuracy between right and left sides can arise from non-compliance to this recommendation.^16^

The slits cut on the inner tray represent another advantage of this technique. As observed by Wendl et al,^15^ problems with bonding can arise from stress caused to the adhesive interface during transfer tray removal. The slits add flexibility to the tray, thus facilitating its removal without trauma. This way, excessive forces over brackets are avoided while the adhesive has not yet reached its peak bond strength. Therefore this implies that methods with excessive retention between tray and brackets can be undesirable, and while reducing tray extension favors its removal, it may lead to undesirable displacement during bracket transfer. However, due to diminished bracket-tray retention, it is not advisable to employ the described technique using small sized brackets, or brackets with little wing retention, such as some esthetic varieties, especially when it comes to mandibular incisors. In this situation, bracket displacement from the tray is likely to occur. 

An additional factor contributing to technique efficiency is the clear tray. It not only allows visual confirmation of fitting and bracket position at the moment of transference, but also permits the use of light-curable material. The latter provides higher initial bond strength than self-curing materials, an asset at the moment of tray removal and immediate insertion of orthodontic archwires.^21^ In addition, it provides enough time for correct tray fitting,^21^ since curing only starts upon activation by the operator. 

Light and self-curable adhesives have been specifically developed for indirect bonding and are commercially available. However, clinical experience and experimental studies have shown Transbond XT system provide excellent results when associated to this technique. The study by Shimizu et al^13^ supports this finding by stating that Sondhi Rapid Set and Transbond XT Primer systems displayed similar shear bond strength after the indirect method was employed.

Simplicity, accuracy and reproducibility of this technique lead to its efficiency as an orthodontic bonding method, providing the advantages related to indirect bonding to benefit both professionals and patients involved in this process. 

## FINAL CONSIDERATIONS

The indirect bonding technique is a better method when it comes to precision in placing brackets. However, in order to be successful, the technique must offer sufficient criteria that allow this advantage to be achieved. By judiciously following the steps described herein, it is possible to carry out the procedure with adequate precision and efficiency.
